# Supplementary material for: Discriminating cross-reactivity in polyclonal IgG1 responses against SARS-CoV-2 variants of concern
Source: Nat Commun. 2022 Oct 15;13:6103. doi: 10.1038/s41467-022-33899-1 (PMC9568977; doi:10.1038/s41467-022-33899-1)
Supplement: Supplementary file 1 — Supplementary Information [file 41467_2022_33899_MOESM1_ESM.pdf]

Supplementary information to:

Discriminating cross-reactivity in polyclonal IgG1 responses against SARS-CoV-2 variants of concern

Danique M.H. van Rijswijck[1,2,‡], Albert Bondt[1,2,‡], Max Hoek[1,2], Karlijn van der Straten[3,4], Tom G. Caniels[3], Meliawati Poniman[3], Dirk Eggink[5], Chantal Reusken[5], Godelieve J. de Bree[4], Rogier W. Sanders[3], Marit J. van Gils[3], Albert J.R.Heck[1,2,*]

[1]Biomolecular Mass Spectrometry and Proteomics, Bijvoet Center for Biomolecular Research and Utrecht Institute for Pharmaceutical Sciences, University of Utrecht, Padualaan 8, Utrecht 3584 CH, the Netherlands
[2]Netherlands Proteomic Center, Padualaan 8, Utrecht 3584 CH, the Netherlands
[3]Department of Medical Microbiology, Amsterdam UMC, University of Amsterdam, Amsterdam Institute for Infection and Immunity, Meibergdreef 9, Amsterdam 1105 AZ, the Netherlands.
[4]Department of Internal Medicine, Amsterdam UMC, Vrije Universiteit Amsterdam, Amsterdam Institute for Infection and Immunity, Meibergdreef 9, Amsterdam 1105 AZ, the Netherlands.
[5]National Institute for Public Health and the Environment, RIVM, Antonie van Leeuwenhoeklaan 9, 3721 MA Bilthoven, the Netherlands

[‡]These authors contributed equally
[*]Corresponding Author: Albert J.R. Heck, E-mail: a.j.r.heck@uu.nl

Supplementary Table 1| Plasma donor characteristics. With the Severity score of the World Health Organization (WHO) based on hospitalization, intensive care unit (ICU) admission, immune suppression symptoms and treatment. With Score 1: no limitations of activities, Score 2: limitations of activities, Score 3: hospitalized, but no oxygen therapy, Score 4: hospitalized with oxygen therapy by mask or nasal cannulae.

Record Id	Proven PCR test of infection	Variant of infection	Age	Gender	Severity score WHO	Respiratory symptoms 1=Yes; 2=No	Fever 1=Yes; 2=No	Hospital admission 1=Yes; 2=No	ICU 1=Yes; 2=No	Duration admission in days	Chronic immune suppresion 1=Yes; 2=No	Short immune suppresion during COVID-19?	Treatment during hospital admission?	Date when COVID-19 symptoms started	Date of blood withdrawal	Days between symptoms and blood withdrawal
**002**	Yes	WT	44	Female	2	1	1	2	2	0	No	No		2020-02-25	2020-03-23	27
**003**	Yes	WT	69	Male	4	1	1	1	1	13	No	No	Cefotaxim, Ciproflox	2020-03-08	2020-03-23	15
**303**	Yes	α	66	Male	2	1	1	2	2	0	No	No		2021-01-08	2020-02-04	27
**304**	Yes	α	59	Female	2	1	1	2	2	0	No	No		2021-01-02	2020-02-04	33
**307**	Yes	β	18	Female	1	1	1	2	2	0	No	No		2021-01-13	2021-02-12	30
**308**	Yes	β	18	Male	1	1	1	2	2	0	No	No		2021-01-07	2021-02-12	36
**309**	Yes	γ	32	Female	1	1	2	2	2	0	No	No		2021-01-09	2021-02-19	41
**310**	Yes	γ	37	Male	1	1	2	2	2	0	No	No		2021-01-12	2021-02-19	38

Supplementary Table 2| The total amount of S-protein directed IgG1 clones, and their concentrations vary substantially in between donors. For each donor and each variant, the number of IgG1 clones detected binding to each VOC S-protein-trimer mutant is provided and compared to the total number of IgG1 clones detected in full plasma (bottom rows). Making use of the two recombinant IgG1 mAbs internal standards, we could also estimate the total and individual concentrations of IgG1s. As shown in the table the total concentration of IgG1s in these eight donors varies in between 100 ug/ml to 582 ug/ml. The total concentrations of IgG1s binding to the VOC S-protein-trimer mutants, varies much more from 0.1 ug/ml to 31,6 mg/mL.

	**Record ID**	**002**	**003**	**303**	**304**	**307**	**308**	**309**	**310**
	**VOC of infection**	**WT**	**WT**	**α**	**α**	**β**	**β**	**γ**	**γ**
	**Score**	**2**	**4**	**2**	**2**	**1**	**1**	**1**	**1**
**WT**	**# of clones**	46	192	277	13	2	5	71	23
	**Concentration (μg/mL)**	0.6	21.7	31.6	0.6	<0.1	0.3	2.3	0.3
**α**	**# of clones**	9	116	281	19	1	4	50	27
	**Concentration (μg/mL)**	0.1	12.3	14.6	0.7	<0.1	0.1	0.8	0.3
**β**	**# of clones**	9	68	204	6	3	8	39	18
	**Concentration (μg/mL)**	0.1	7.0	10.1	0.6	<0.1	0.3	1.2	0.3
**γ**	**# of clones**	7	114	208	11	3	5	82	33
	**Concentration (μg/mL)**	0.2	6.7	9.5	0.9	<0.1	0.3	3.1	0.5
**Full plasma**	**# of clones**	381	370	517	247	363	386	454	284
	**Concentration (μg/mL)**	356.9	582.1	317.8	100.3	219.1	272.8	340.7	153.7

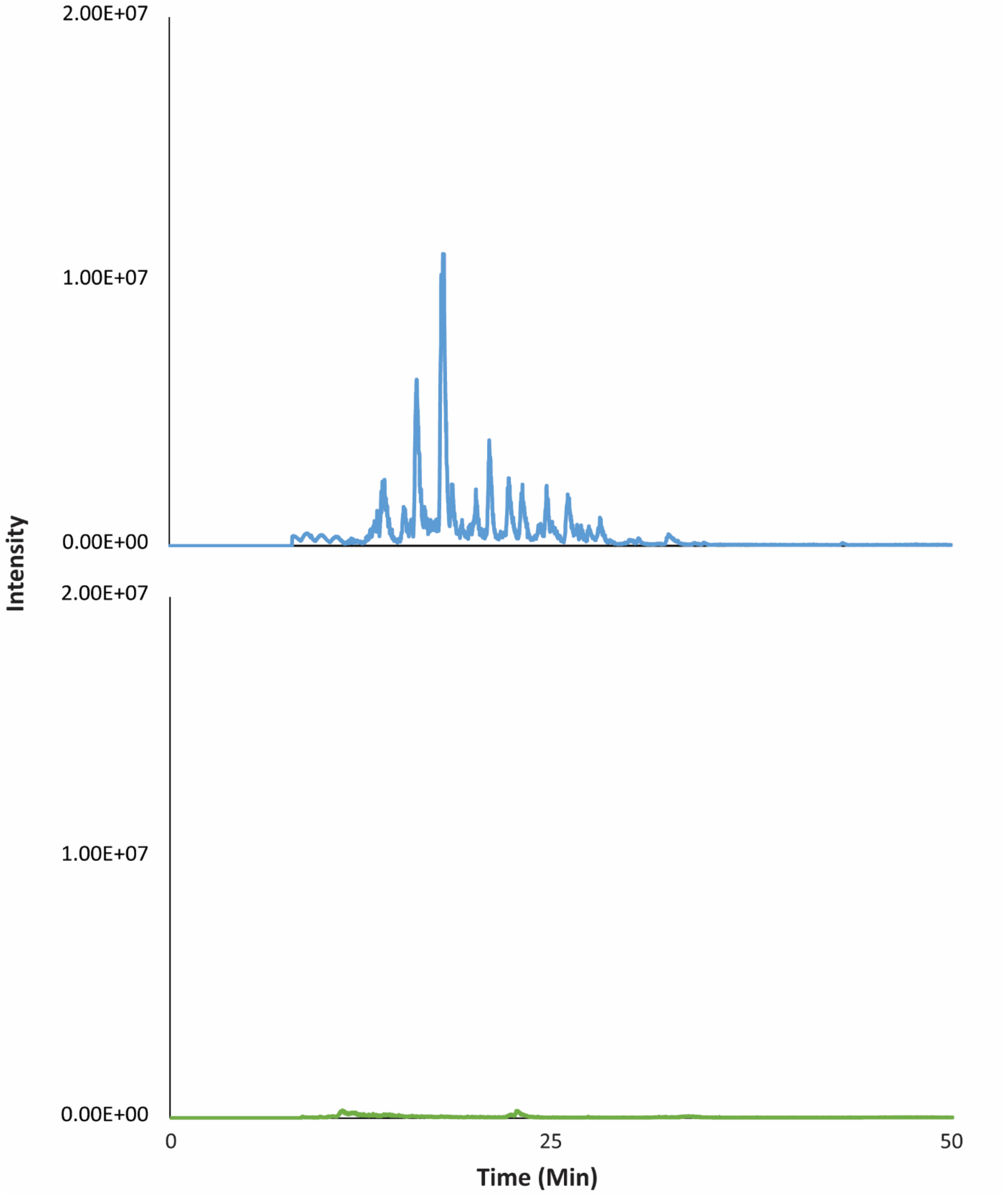

Supplementary Fig. 1| Evaluation of non-specific binding to the NHS agarose beads. LC-MS chromatograms obtained for the bound (green) and unbound (blue) fraction when using plasma of donor 003 and NHS agarose beads without any spike attached ('bare' NHS agarose beads). In the bound LC-MS trace no Fab related peaks were detected, indicating that there is no substantial non-specific binding to the agarose beads.

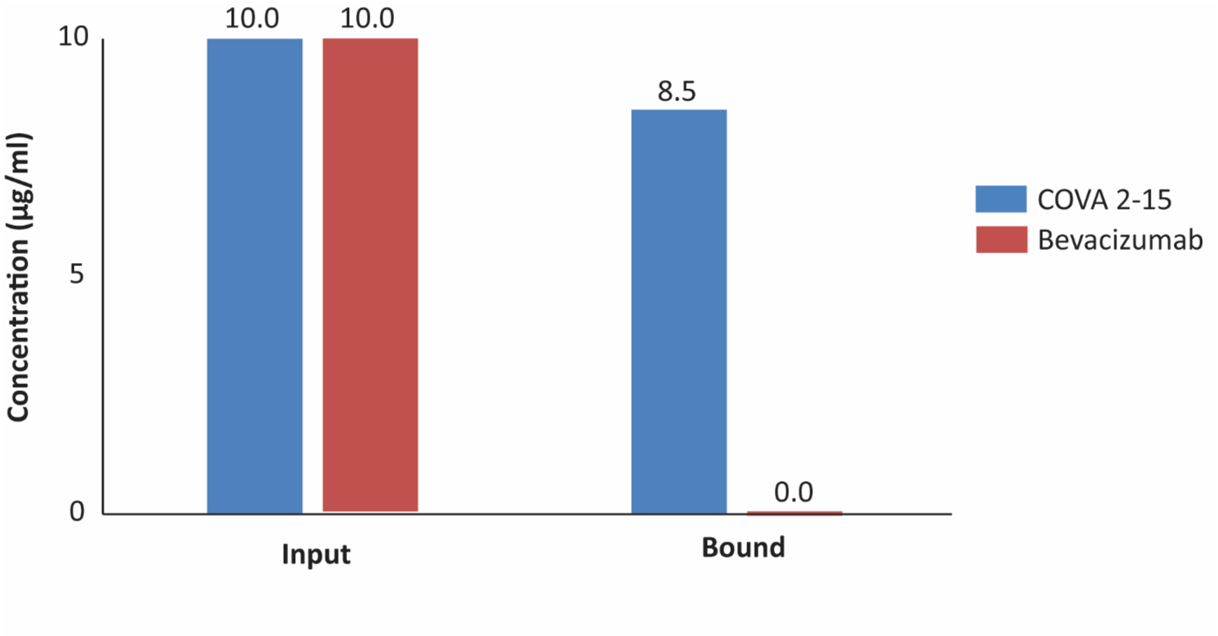

Supplementary Fig. 2| Assessing bead specificity by spiking in an anti-spike IgG1 monoclonal (COVA 2-15) and an anti-VEGF IgG1 monoclonal (Bevacizumab) as control. We used the WT S-protein loaded beads and plasma of Donor 003 in which we spiked 10 µg/ml COVA 2-15 IgG1 (Brouwer et al. Science 2022) and 10 µg/ml Bevacizumab (anti-VEGF). The analysis revealed that about 85% of the COVA 2-15 IgG1 bound to the beads and could be recovered, while no detectable amount of Bevacizumab was retrieved following the same procedure.

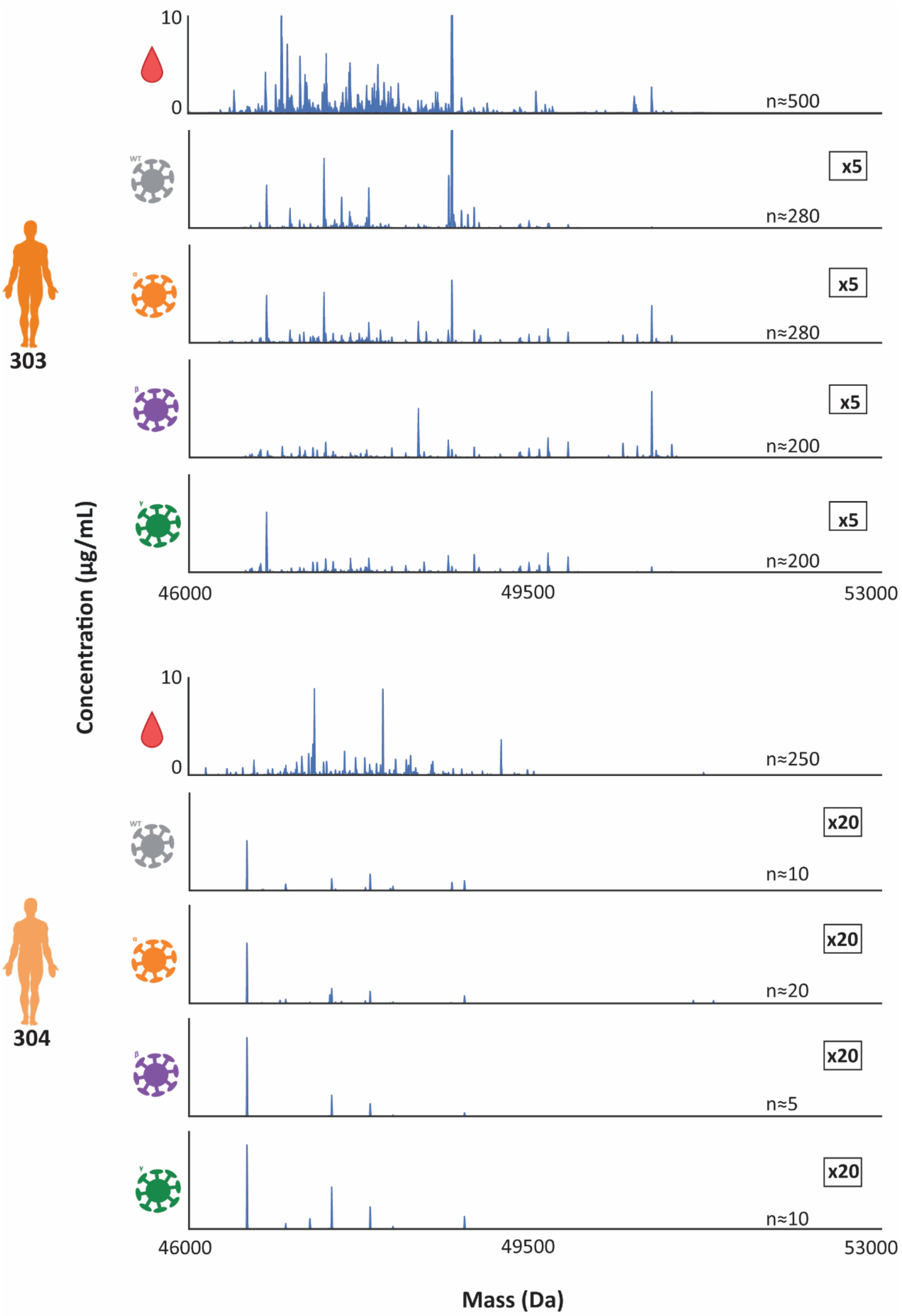

Supplementary Fig. 3| Fab mass profiles for donor 303 and 304. From top to bottom, the full plasma Fab profile, followed by the S-protein directed Fab profiles using the WT, Alpha, Beta and Gamma VOC variant. Each peak represents a unique Fab at its detected mass and plasma concentration. In each plot the number of unique Fab identified is indicated. The number on the right of each S-protein directed Fab profile, shows the magnification of the y-axis, compared to the data for the full plasma Fab profile.

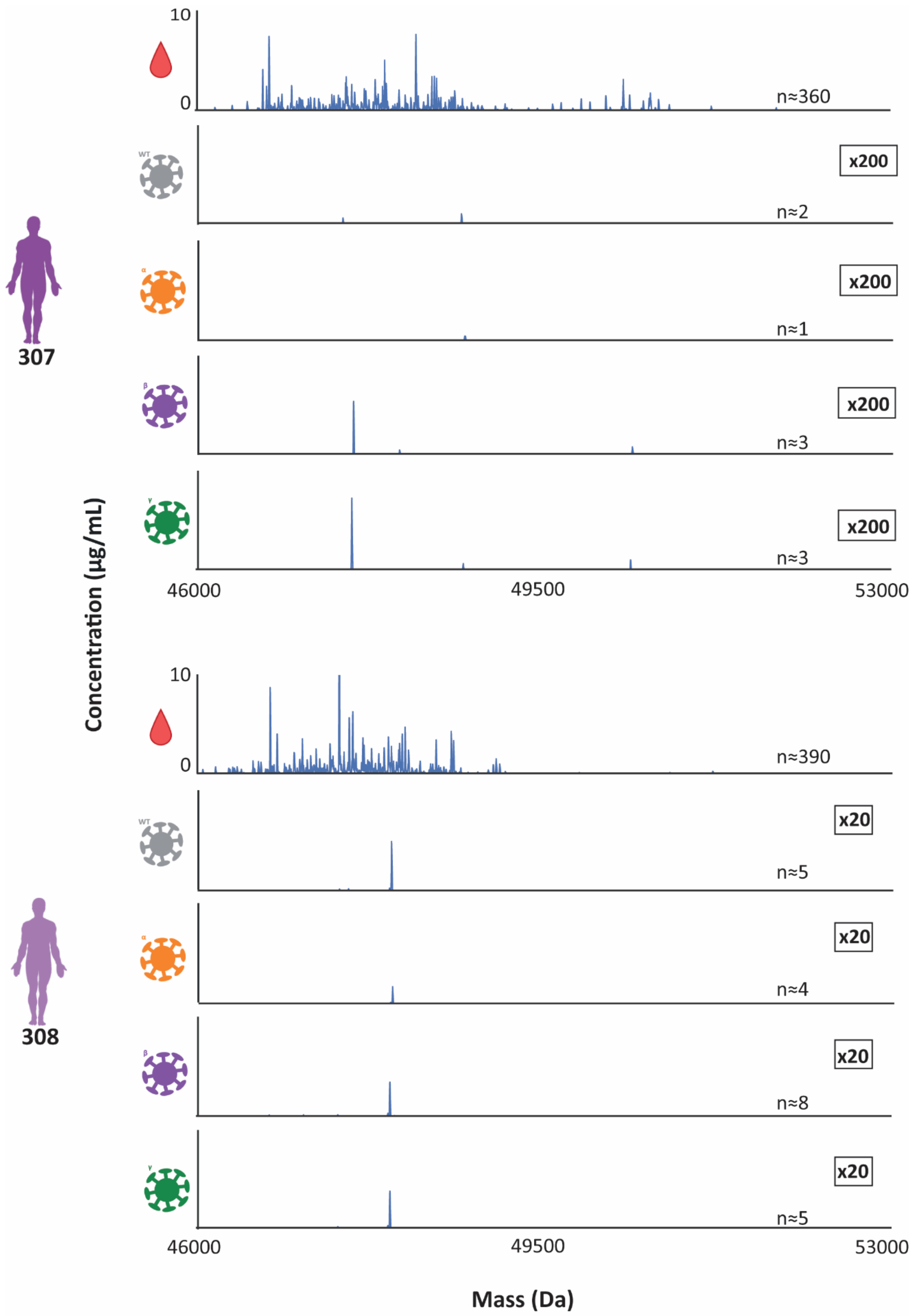

Supplementary Fig. 4| Fab mass profiles for donor 307 and 308. From top to bottom, the full plasma Fab profile, followed by the S-protein directed Fab profiles using the WT, Alpha, Beta and Gamma VOC variant. Each peak represents a unique Fab at its detected mass and plasma concentration. In each plot the number of unique Fab identified is indicated. The number on the right of each S-protein directed Fab profile, shows the magnification of the y-axis, compared to the data for the full plasma Fab profile.

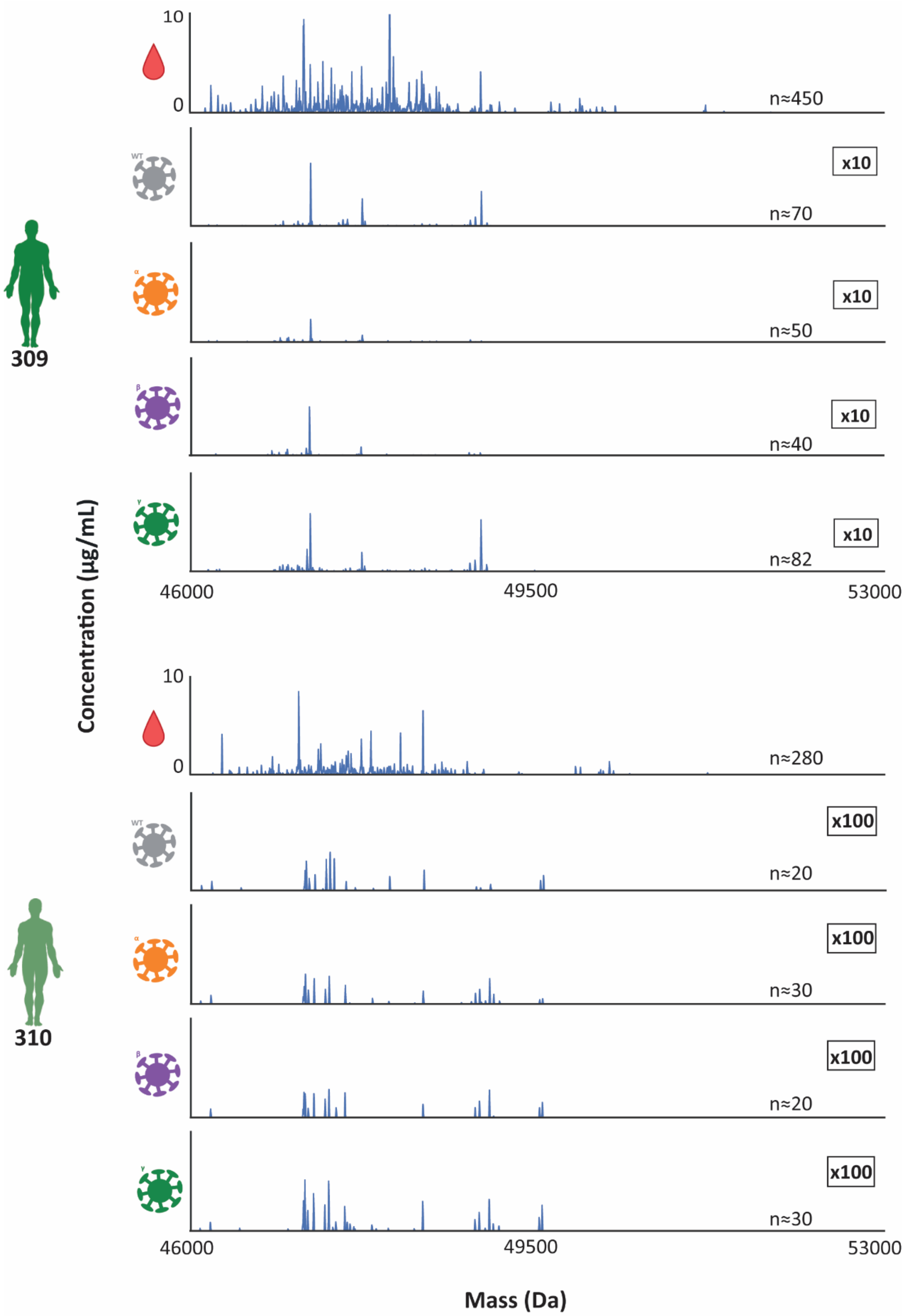

Supplementary Fig. 5| Fab mass profiles for donor 309 and 310. From top to bottom, the full plasma Fab profile, followed by the S-protein directed Fab profiles using the WT, Alpha, Beta and Gamma VOC variant. Each peak represents a unique Fab at its detected mass and plasma concentration. In each plot the number of unique Fab identified is indicated. The number on the right of each S-protein directed Fab profile, shows the magnification of the y-axis, compared to the data for the full plasma Fab profile

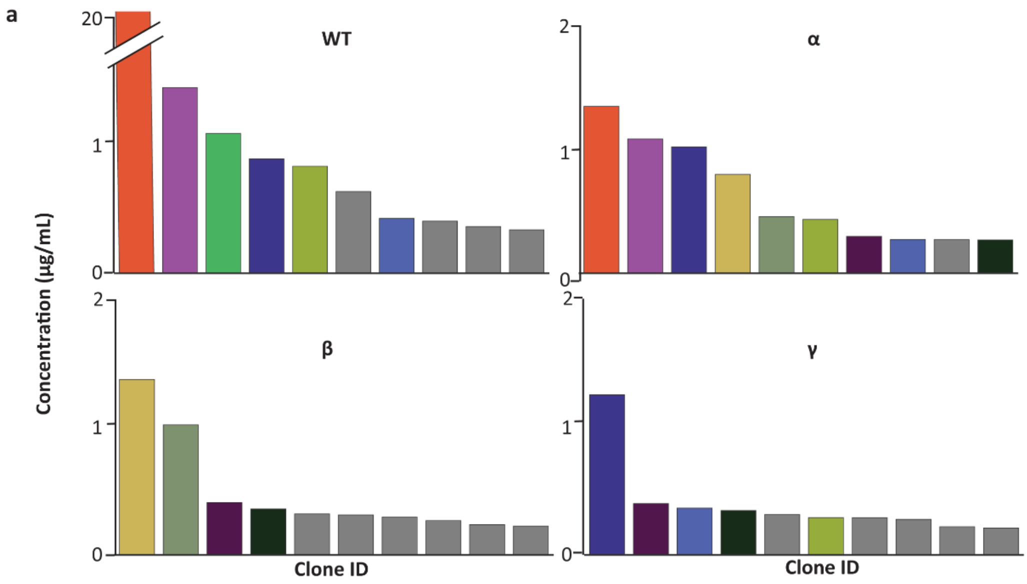

b

Clone ID (RT (min) # Mass (Da))	WT (μg/mL)	α (μg/mL)	β (μg/mL)	γ (μg/mL)
20.1 **449** 48,655.2	18.1	1.3	0.2	0.1
18.4 **208** 47,361.0	1.4	1.0	N.D.	N.D.
18.0 **124** 46,780.0	0.9	1.0	N.D.	1.2
24.9 **17** 50,677.2	0.1	0.8	1.3	0.1
27.2 **442** 48,315.6	0.1	0.4	1.0	N.D.
15.0 **354** 47,814.5	0.8	0.4	N.D.	0.3
22.2 **387** 49,627.9	0.1	0.3	0.4	0.4
18.0 **483** 48,880.5	0.4	0.3	0.2	0.4
16.3 **456** 48,618.2	0.1	0.3	0.4	0.3
21.6 **455** 48,623.5	1.1	0.1	N.D.	0.3

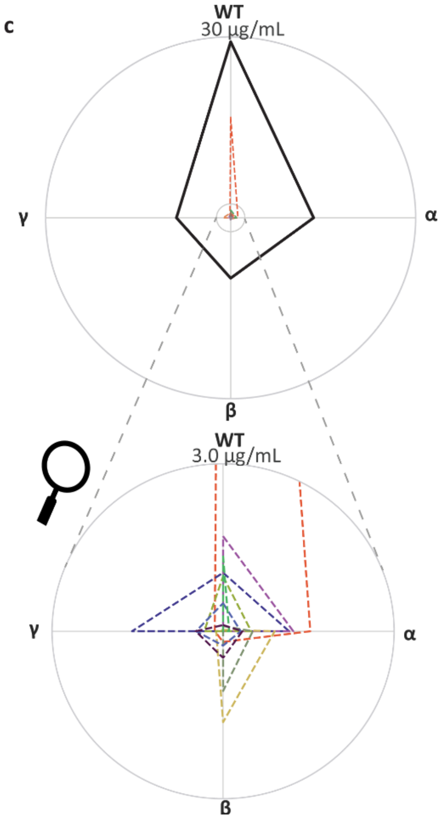

Supplementary Fig. 6| Within a single donor (303) IgG1 clones display distinctive cross-reactivity versus the S-protein variants (A) Quantitative comparison of the ten most abundant IgG1 fab clones enriched from the plasmaof donor 003. Each bar represents one of the 10 most abundant antibody clones with the height indicating concentration in ug/ml. using the same colors for the clones corresponding to panel B. Each clone that was not in the total top 10 but was in the top 10 for that specific VOC is colored grey **(B)** The table depicts the top 10 most abundant clones showing the concentration that in total is enriched using the different S-protein variants for that specific clone. This top 10 was selected based on the clones that in total, by summing up the concentrations found against the different S-protein variants, showed the highest concentrations. The colored bars in (A) and the dotted lines in the radar plot in (C) are corresponding to the clones in the table. **(C)** Radar plot with on each edge one of the tested S-protein variants. These plots depict the difference in binding of specific clones against the different S-protein variants. The thick solid gray line representing the sum of all enriched IgG1 clones against the specific S-protein variants. The color coding is identical as used in (A) and (B), corresponding to the same unique IgG1 clones. The magnifying glass used in this figure was retrieved via Wikipedia.
