# Supplementary material for: Discriminating cross-reactivity in polyclonal IgG1 responses against SARS-CoV-2 variants of concern
Source: Nat Commun. 2022 Oct 15;13:6103. doi: 10.1038/s41467-022-33899-1 (PMC9568977; doi:10.1038/s41467-022-33899-1)
Supplement: Supplementary file 3 — Reporting Summary [file 41467_2022_33899_MOESM3_ESM.pdf]

nature portfolio

Corresponding author(s): Albert J R Heck

Last updated by author(s): Sep 15, 2022

Reporting Summary

Nature Portfolio wishes to improve the reproducibility of the work that we publish. This form provides structure for consistency and transparency in reporting. For further information on Nature Portfolio policies, see our Editorial Policies and the Editorial Policy Checklist.

Statistics

For all statistical analyses, confirm that the following items are present in the figure legend, table legend, main text, or Methods section.

n/a	Confirmed	
☒	☐	The exact sample size (*n*) for each experimental group/condition, given as a discrete number and unit of measurement
☒	☐	A statement on whether measurements were taken from distinct samples or whether the same sample was measured repeatedly
☒	☐	The statistical test(s) used AND whether they are one- or two-sided<br>*Only common tests should be described solely by name; describe more complex techniques in the Methods section.*
☒	☐	A description of all covariates tested
☒	☐	A description of any assumptions or corrections, such as tests of normality and adjustment for multiple comparisons
☐	☒	A full description of the statistical parameters including central tendency (e.g. means) or other basic estimates (e.g. regression coefficient) AND variation (e.g. standard deviation) or associated estimates of uncertainty (e.g. confidence intervals)
☒	☐	For null hypothesis testing, the test statistic (e.g. *F*, *t*, *r*) with confidence intervals, effect sizes, degrees of freedom and *P* value noted<br>*Give P values as exact values whenever suitable.*
☒	☐	For Bayesian analysis, information on the choice of priors and Markov chain Monte Carlo settings
☒	☐	For hierarchical and complex designs, identification of the appropriate level for tests and full reporting of outcomes
☒	☐	Estimates of effect sizes (e.g. Cohen's *d*, Pearson's *r*), indicating how they were calculated

Our web collection on statistics for biologists contains articles on many of the points above.

Software and code

Policy information about availability of computer code

Data collection	BioPharmaFinder 3.2 (Thermo Scientific)
Data analysis	Python 3.8.3

For manuscripts utilizing custom algorithms or software that are central to the research but not yet described in published literature, software must be made available to editors and reviewers. We strongly encourage code deposition in a community repository (e.g. GitHub). See the Nature Portfolio guidelines for submitting code & software for further information.

Data

Policy information about availability of data

All manuscripts must include a data availability statement. This statement should provide the following information, where applicable:

- Accession codes, unique identifiers, or web links for publicly available datasets
- A description of any restrictions on data availability
- For clinical datasets or third party data, please ensure that the statement adheres to our policy

The mass spectrometry data and the data analysis code have been deposited to the MassIVE repository (https://massive.ucsd.edu/ProteoSAFe/static/massive.jsp) with the dataset identifier MSV000089833.

Field-specific reporting

Please select the one below that is the best fit for your research. If you are not sure, read the appropriate sections before making your selection.

- [x] Life sciences
- [ ] Behavioural & social sciences
- [ ] Ecological, evolutionary & environmental sciences

For a reference copy of the document with all sections, see nature.com/documents/nr-reporting-summary-flat.pdf

Life sciences study design

All studies must disclose on these points even when the disclosure is negative.

Sample size	In total 40 different plasma IgG1 clonal repertoires using LC-MS were recorded. Of which 8 were full plasma repertoires (one per donor, in total eight donors), and 8*4 repertoires were obtained from the S-protein variant (in total 4 different variants) directed sub-pools of the IgG's of the different donors. No further sample size calculations was performed, the sample size was determined based on availability.
Data exclusions	No data was excluded.
Replication	Method reproducibility was assessed in previous work (https://doi.org/10.1016/j.cels.2021.08.008). We spiked in two different mAbs (Alemtuzumab and Trastuzumab) in all our measured samples and we ensured that we saw those back in all samples at similar retention times , masses and intensities. This was the case so with this we could confirm that the attempts at replication were successful.
Randomization	No randomization was applicable since there was no organization in experimental groups.
Blinding	No blinding was applicable since there was no organization in experimental groups

Reporting for specific materials, systems and methods

We require information from authors about some types of materials, experimental systems and methods used in many studies. Here, indicate whether each material, system or method listed is relevant to your study. If you are not sure if a list item applies to your research, read the appropriate section before selecting a response.

Materials & experimental systems

n/a	Involved in the study	
[ ]	[x]	Antibodies
[ ]	[x]	Eukaryotic cell lines
[x]	[ ]	Palaeontology and archaeology
[x]	[ ]	Animals and other organisms
[ ]	[x]	Human research participants
[x]	[ ]	Clinical data
[x]	[ ]	Dual use research of concern

Methods

n/a	Involved in the study	
[x]	[ ]	ChIP-seq
[x]	[ ]	Flow cytometry
[x]	[ ]	MRI-based neuroimaging

Antibodies

Antibodies used	Trastuzumab, Roche, Penzberg, Germany, N/A. Alemtuzumab, Genmab, Utrecht, The Netherlands, N/A. Goat-anti-human IgG-PE, Southern Biotech, Cat#:2040-09,RRID:AB_2795648
Validation	All samples included both monoclonal Antibodies. Mass and retention time were detected as expected.

Eukaryotic cell lines

Policy information about cell lines

Cell line source(s)	HEK293F (Invitrogen, cat no. R79009), HEK293T (ATCC, CRL-11268)
Authentication	The cell lines used were not authenticated.
Mycoplasma contamination	All cell lines tested negative for mycoplasma.
Commonly misidentified lines (See ICLAC register)	No commonly misidentified cell lines were used in this study.

Human research participants

Policy information about studies involving human research participants

Population characteristics	Plasma was collected through the COVID-19 Specific Antibodies (COSCA) study. Participents were aged between 18 and 69 years, whereof 4 male and 4 female.
Recruitment	Participants were recruited via the National Institute for Public Health and the Environment. The risk of recruitment bias is deemed low, as potential participants were identified via the Dutch national SARS-CoV-2 sequence surveillance that test PCR swabs in the Netherlands randomly for the presence of VOCs. In case potential participants were interested in participating, contact information was shared with the study team of the COSCA study and a visit was scheduled with the participants.
Ethics oversight	The study participants were included after signing informed consent. The COSCA study was conducted at the Amsterdam University Medical Centre, location AMC, the Netherlands and approved by the local ethical committee of the AMC (NL 73281.018.20).

Note that full information on the approval of the study protocol must also be provided in the manuscript.
